# Evaluation of gingival biotype and bone thickness in maxillary implant patients: A clinical study based on cone-beam computed tomography

**DOI:** 10.34172/japid.026.3716

**Published:** 2026-03-16

**Authors:** Motahare Damavandi, Razieh Azizian, Narges Shojaei

**Affiliations:** ^1^Department of Periodontics, School of Dentistry, Mashhad University of Medical Sciences, Mashhad, Iran; ^2^Department of Periodontology, Faculty of Dentistry, Ilam University of Medical Sciences, Ilam, Iran

**Keywords:** Alveolar bone loss, Computed tomography, Dental implants, Gingiva, Maxillary bone

## Abstract

**Background.:**

The relationship between gingival biotype (GB) and bone thickness is paramount for optimal maxillary implant outcomes. This study aimed to determine the correlation between GB and buccal bone thickness (BBT) and buccal bone height (BBH) in candidates for immediate maxillary implant placement using cone-beam computed tomography (CBCT).

**Methods.:**

This cross-sectional study assessed 54 patients from the Periodontology Department at Ilam University of Medical Sciences Dental School. Gingival thickness (GT), buccal bone measurements (BBT and BBH), and clinical parameters, including keratinized gingival width (KGW) and papillary height (PH), were recorded using Michigan probes and CBCT scans. Statistical analyses (Mann-Whitney and Kruskal-Wallis tests) were conducted to evaluate associations between variables, with *P*<0.05 considered significant.

**Results.:**

Of the participants, 62.96% had a thick GB and 37.04% had a thin GB. Thin biotypes were significantly more prevalent among females (*P*<0.05). The thick GB group exhibited significantly greater KGW and overall mean BBT compared to the thin GB group (*P*<0.05). Conversely, no statistically significant differences were observed between thick and thin biotypes regarding overall mean BBH or PH (*P*>0.05).

**Conclusion.:**

A thick gingival biotype is anatomically associated with greater buccal bone thickness and keratinized gingival width. However, there were no statistically significant differences in buccal or papillary bone height between the different gingival biotypes. Thin gingival biotypes are more prevalent among females.

## Introduction

 Gingival biotype (GB) refers to defining the thickness of keratinized gingiva in the buccolingual dimension.^1^ It plays a pivotal role in maintaining periodontal health through the overall anatomy of the periodontium.^2^ Gingival thickness (GT) is determined by the shape and size of the tooth root and the contour of the alveolar bone.^3^ This parameter is typically categorized into two main biotypes: the thick tissue biotype, with a thickness exceeding 2 mm, and the thin tissue biotype, with a thickness less than 1.5 mm.^4^ In thin biotypes, the teeth generally exhibit a triangular shape with shorter proximal contacts, whereas in thick biotypes, the teeth are usually more square-shaped.^1^

 Keratinized gingiva plays a vital role in providing a stable foundation for both dental and aesthetic treatments.^5^ In areas where keratinized mucosa is reduced, rapid loss of attachment and bone stability can occur.^6^ The interdental papilla occupies a critical area and significantly influences both the contour and health of the tissue during aesthetic procedures. Loss of papillary height (PH) can lead to an unaesthetic appearance, difficulties with food intake, and speech issues.^7,8^ The appearance of the papilla is influenced by several factors, including age, gender, tooth shape, proximal contact height, bone height, periodontal disease, orthodontic treatment history, and interproximal gingival thickness.^9^ Fischer et al.^10^ reported that young men tend to have thicker gingiva compared to women. Additionally, a thicker gingival phenotype was more commonly observed in the maxillary region, while the mandibular region exhibited a higher prevalence of a thin gingival phenotype. Some studies have indicated a positive correlation between keratinized gingival width (KGW) and GT. Furthermore, biotype has been identified as a significant factor influencing papilla height.^11,12^ The findings of Vlachodimou et al.^13^ revealed that thin gingival biotypes, particularly those with a keratinized gingival width of < 2 mm and a gingival thickness of < 0.5 mm, are more susceptible to gingival recession and periodontal issues. This relationship between gingival phenotype and KGW plays a fundamental role in determining periodontal treatment plans and ensuring long-term tissue stability​.

 Recently, immediate implant placement has gained attention as an innovative and efficient approach for treating the mid-buccal area of the maxilla, offering several advantages, including reduced surgical procedures, shorter treatment times, higher patient acceptance, decreased psychological stress, and enhanced aesthetics.^14,15^ However, to improve the success of this method, a more detailed understanding of the GB and the underlying bone thickness in the mid-buccal region is essential. The GB and underlying bone thickness are key factors that affect the success of immediate implant placement in the maxillary region. Patients with different gingival biotypes (GBs) exhibit varying responses to inflammation and periodontal treatment. Da Silva et al.^16^ reported that the gingival phenotype (thin or thick) can indirectly affect the success of immediate implant placement. They noted that patients with different gingival types, particularly those with thin gingiva, exhibit varying responses to inflammation and periodontal treatments. Therefore, recognizing these characteristics is critical when performing periodontal, prosthetic, aesthetic, and implant treatments.^17,18^ Gingival recession, particularly in patients with a thin or scalloped GB, is a significant concern. In this context, Khursheed et al.^19^ highlighted that gingival recession is one of the most common mucogingival defects, which may occur alone or in combination with issues such as thin gingival biotypes, shallow vestibule, high frenal attachment, and cervical dental steps. In contrast, thick GBs are more resistant to trauma, are more likely to form periodontal pockets, and exhibit fibrotic responses, whereas thin biotypes are more prone to recession and shrinkage.^20^ Supporting this observation, Koppolu et al.^21^ demonstrated that the thick gingiva biotype is associated with good periodontal health and the ability to resist trauma and recession. In contrast, thin gingiva is more predisposed to recession compared to thick gingiva.

 The GB is influenced by tissue responses to physical and chemical trauma, outcomes of periodontal and implant surgeries, and orthodontic or restorative treatments.^22^ In this context, the thickness of keratinized gingiva plays a crucial role in maintaining periodontal health.^23^ Gingival width is also a critical indicator in periodontal evaluations, influencing decisions regarding the necessity and selection of surgical techniques.^24^ Therefore, a precise analysis of the relationship between GB and underlying bone thickness in the mid-buccal region is of significant importance.

 In recent years, the digitalization of dental technologies has introduced a wide range of methods for assessing and correlating anatomical structures within and around the oral and maxillofacial system. These methods include features that are not detectable through conventional examination.^25^ Techniques such as direct probing, probe transparency technique, ultrasonic devices, computed tomography (CT) scans, and cone-beam computed tomography (CBCT) are now available.^26^ In the probe transparency technique, if the tip of the periodontal probe is visible through the gingiva during probing, the gingiva is classified as thin.^27^ This simple, highly reproducible, noninvasive method is commonly used to determine GB.^28^ The CBCT offers several advantages over traditional CT scans, with the most notable being lower radiation exposure to the patient.^29^ This technique provides detailed information about both hard and soft tissues, facilitating implant placement. With its three-dimensional imaging capabilities, CBCT offers greater diagnostic accuracy and more comprehensive analysis compared to two-dimensional imaging.^30^

 The aesthetic zone in the anterior maxilla typically extends from the first premolar on one side to the first premolar on the other side.^31^ However, in some individuals, this zone may extend to the mesial aspect of the first molar. Although this area is of critical aesthetic importance, limited research exists on bone resorption following tooth extraction or bone thickness in the premolar region among the Iranian population.^32^ Most existing studies have focused on orthodontic perspectives, primarily aiming to determine the bone thickness required for mini-screws to provide anchorage and facilitate tooth movement in this area.^33^ However, fewer studies have investigated the bone thickness necessary for immediate implant placement, specifically regarding the minimum required thickness for such treatments in this region. Therefore, given the importance of GB and the underlying bone in immediate implant placement, this study aimed to determine the correlation between GB, BBT, and height in candidates for immediate maxillary implant placement using CBCT analysis.

## Methods

###  Sample Size and Patient Recruitment

 This cross-sectional, descriptive, epidemiological study was conducted on 54 patients who visited the Periodontology Department at the Ilam University of Medical Sciences Dental School during 2023‒2024. Written informed consent was obtained from patients who were candidates for immediate implant placement, and the study was approved by the Ethics Committee of Ilam University of Medical Sciences (IR.MEDILAM.REC.1402.055). The study procedures were thoroughly explained to each patient before the study began. Statistical methods were employed to analyze the relationship between GT and bone measurements. The required sample size was estimated to be 54 individuals, based on the study by Shah et al.,^34^ considering a margin of error (d) of 0.22, a standard deviation (σ) of 0.82, and a confidence level (1-α) of 0.95 (Equation 1).

$$N=\frac{Z_{1\frac{\alpha }{2}}{}^{2}\times \delta^{2}}{d^{2}}=\frac{{\left(1.96\right)}^{2}\times {\left(0.82\right)}^{2}}{{\left(0.22\right)}^{2}}=53.36≅54$$

###  Inclusion and Exclusion Criteria

 The inclusion criteria consisted of participants aged 18–50 years with maxillary incisors indicated for immediate implant placement. Periodontal health was confirmed by the absence of inflammation, probing depths < 3 mm, and bleeding on probing (BOP) < 30%. Systemic conditions were evaluated based on each patient’s medical history. Following tooth extraction, the socket was required to be intact with preserved buccal bone and a minimum of 4 mm of apical bone height.

 The exclusion criteria included a history of periodontal surgery in the anterior maxilla, systemic diseases or medications influencing periodontal status, probing depths ≥ 3 mm, attachment loss or gingival enlargement at the central incisors, previous orthodontic treatment of these teeth, traumatic injuries deforming the incisors, pregnancy or breastfeeding, high labial frenum attachment, interproximal or cervical caries or restorations, smoking, crowding, or mouth breathing. All patients received oral hygiene instructions one week before parameter measurements, and scaling was performed at least four weeks before the measurements if necessary.^32^

###  Clinical Parameters

**Figure 1:**
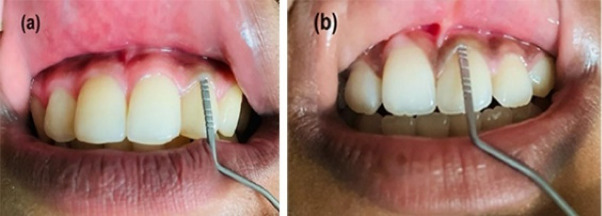
Clinical assessment of gingival thickness using the transgingival probing method, illustrating (a) a thick gingival biotype ( > 1 mm) and (b) a thin gingival biotype ( ≤ 1 mm)

 In this study, gingival thickness (GT) was measured using the transgingival probing method, whereas keratinized gingival width (KGW), probing depth, and papillary height (PH) were assessed using a periodontal probe. A single calibrated examiner performed all measurements. Accordingly, GT was assessed after local anesthesia with 2% lidocaine, and a Michigan periodontal probe was inserted perpendicularly into the mid-facial buccal gingiva 2 mm apical to the gingival margin until gentle contact with the underlying bone was achieved. Gingival biotype classification was based on the measured thickness, with values > 1 mm classified as thick and values ≤ 1 mm classified as thin (Figure 1).^35^

 In addition, KGW was defined as the mean distance from the gingival margin to the mucogingival junction at the mid-facial region of the maxillary central incisors.^13^ Probing depth was measured as the average depth of the probe inserted into the gingival sulcus at the mid-facial region of the maxillary central incisors.^36^ Finally, PH was the distance from the tip of the papilla to a line connecting the gingival zenith of the adjacent teeth. The average measurement was reported for the three papillae between the central incisors and their distal papillae.^37^

###  CBCT Acquisition and Measurements

 All CBCT examinations were performed using an HDXWill CBCT system (HDXWill, South Korea). Image acquisition was conducted using a standardized protocol applied uniformly to all participants. The acquisition parameters included a tube voltage of 85 kV, a tube current of 7 mA, a slice thickness of 0.3 mm, and a maximum field of view (FOV) of 16 × 9 cm. The voxel size was set at 0.2 mm for all scans. CBCT images were analyzed using the manufacturer-provided software. All measurements were referenced to the alveolar crest. BBT was assessed perpendicular to the long axis of the tooth, whereas BBH was defined as the vertical distance from the alveolar crest to the most coronal point of the buccal bone.

###  Data Collection

 The required information, including age, gender, GB, GT, and bone height, width, and thickness, was collected using a structured checklist.

###  Statistical Analysis

 Statistical analysis was performed using SPSS 16.0 (IBM SPSS Statistics, USA) and Stata 10.3.1 (StataCorp). Data normality was assessed using the Shapiro–Wilk test. As the variables did not follow a normal distribution, non-parametric statistical tests were applied. Descriptive statistics were reported as mean ± standard deviation (SD) for quantitative variables. The chi-square test was used to evaluate the association between GT and gender. Comparisons of quantitative variables between gingival biotype groups were performed using the Mann–Whitney U test, while the Kruskal–Wallis test was applied where appropriate. A *P* value of < 0.05 was considered statistically significant for all analyses.

## Results

**Figure 2:**
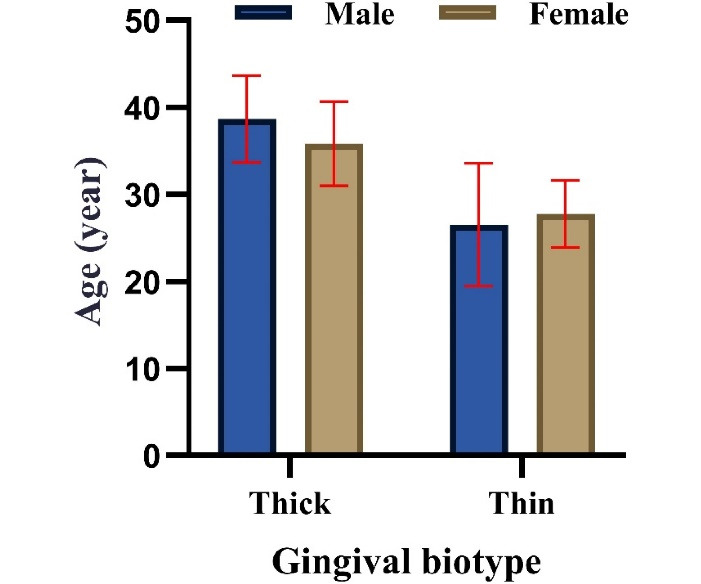
Mean age of male and female participants according to gingival biotype (thick and thin)

 The results of the present study revealed that 34 participants (62.96%) had a thick GT, while 20 participants (37.04%) had a thin GT. Statistical analysis revealed a significant association between GT and gender (chi-squared test, *P* < 0.05), with the thin GT being more prevalent in females (76.2% [95% CI: 52.8‒91.8%]) and the thick GT more common in males. The results also indicated that the mean age of males (mean and standard deviation [SD]) (37.15 ± 6.58) was higher than that of females (29.95 ± 5.46). In addition, the mean gingival biotype for thin GB was lower in males (26.50 ± 7.05) compared to females (27.75 ± 3.87), while the mean gingival biotype for thick GB was more pronounced in females (35.83 ± 4.83) than in males (38.69 ± 5.00). These differences were statistically significant (Kruskal-Wallis test, *P* < 0.05, Table 1, Figure 2).

**Figure 3:**
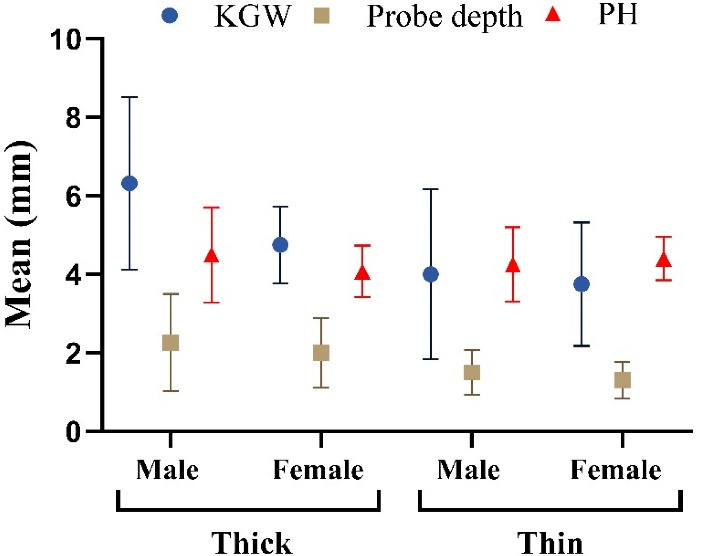
Mean values of keratinized gingival width (KGW), probing depth, and papillary height (PH) in males and females with thick and thin gingival biotypes

 Furthermore, the findings revealed that, for the thin GB, the mean values for KGW, probing depth, and PH in males and females were as follows: 4.00 ± 2.16, 2.26 ± 1.24, 4.25 ± 0.95, 3.75 ± 1.75, 1.31 ± 0.47, and 4.40 ± 0.55 mm, respectively. In contrast, for the thick GB, the corresponding values for males and females were 6.32 ± 2.20, 1.50 ± 0.57, 4.50 ± 1.21, 4.75 ± 0.98, 2.00 ± 0.89, and 4.08 ± 0.66 mm, respectively (Table 1). In this regard, a statistically significant difference in the mean KGW and probing depth was observed between the thick and thin GBs (Mann-Whitney test, *P* < 0.05). However, no significant difference was found in PH (Kruskal-Wallis test, *P* > 0.05, Figure 3).

**Figure 4:**
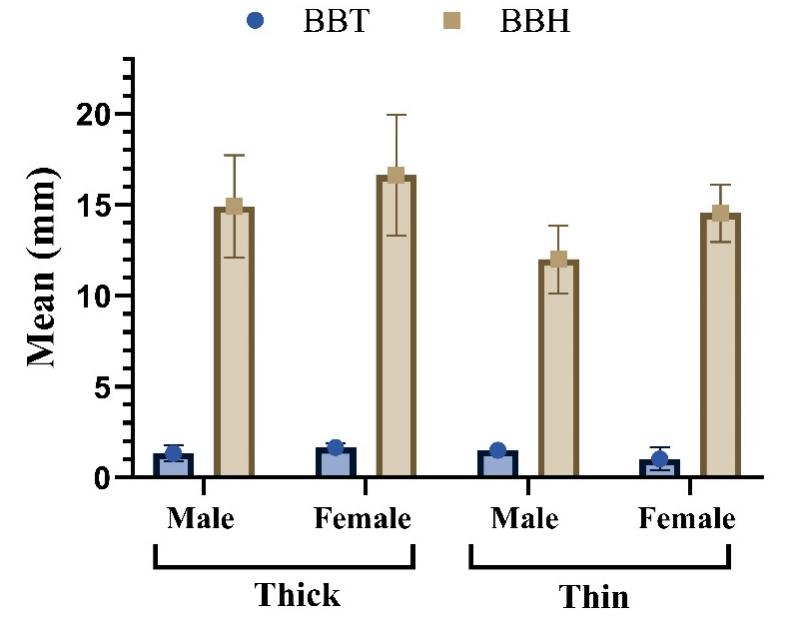
Mean buccal bone thickness (BBT) and buccal bone height (BBH) in males and females with thick and thin gingival biotypes

 The results of measuring buccal bone height (BBH) and thickness in thick and thin GBs showed that the mean BBH was greater in the thick gingival biotype (F = 16.63 and M = 14.91) compared to the thin GB (F = 14.53 and M = 12) (Table 1, Figure 4). However, this difference was not statistically significant (Kruskal-Wallis test, *P* > 0.05). Similarly, the mean BBT in the thick GB was 1.66 mm for females and 1.34 mm for males, while in the thin GB, it was 1.03 mm for females and 1.50 mm for males (Figure 4). This difference between the two groups was statistically significant (Kruskal-Wallis test, *P* < 0.05).

**Table 1:** Comparative analysis of clinical parameters in thin and thick gingival biotypes

**Variable**	**Thick gingival biotype**					**Thin gingival biotype**				
	**Gender**	**Mean**	**SD**	**Min.**	**Max.**	**Gender**	**Mean**	**SD**	**Min.**	**Max.**
Age	F	35.83	4.83	28	41	F	27.75	3.87	22	37
	M	38.69	5.00	27	45	M	26.50	7.05	19	36
KGW	F	4.75	0.98	4	6	F	3.75	1.75	2	7
	M	6.32	2.20	2	11	M	4.00	2.16	2	7
Probe depth	F	2.00	0.89	1	3	F	1.31	0.47	1	2
	M	1.50	0.57	1	2	M	2.26	1.24	0.5	6
PH	F	4.08	0.66	3	5	F	4.40	0.55	3	5
	M	4.50	1.21	1	7	M	4.25	0.95	3	5
BBT	F	1.66	0.25	1.5	2	F	1.03	0.64	0.5	2.5
	M	1.34	0.44	0.5	2	M	1.50	0.00	1.5	1.5
BBH	F	16.63	3.34	12.80	22	F	14.53	1.58	11.50	18
	M	14.91	2.81	9.80	22	M	12.00	1.87	10.00	14.5

KGW: keratinized gingival width, PH: papilla height, BBT: buccal bone thickness, BBH: buccal bone height, F: female, M: male

## Discussion

 In the present study, 62.96% of patients had a thick gingival biotype, while 37.04% had a thin biotype. These findings are similar to those reported by Shao et al.,^28^ who found that 59.68% of the teeth had a thick biotype, while 40.32% had a thin biotype. Similarly, research by Assiri et al.^38^ demonstrated a higher prevalence of the thick biotype than the thin biotype. These studies are consistent with our findings. However, they contrast with García-Cortés et al.,^39^ who reported a higher prevalence of the thin biotype compared to the thick biotype.

 Tissue biotype plays a crucial role in determining the outcomes of dental treatments.^40^ The initial thickness of the gingiva can predict the success of root coverage procedures or restorative treatments.^41^ The GB is a key factor influencing the results of dental procedures. Individuals with a thin biotype tend to experience more gingival recession during non-surgical treatments.^42^ Additionally, those with a thin biotype are more prone to losing attached tissue and experiencing epithelial damage, necessitating non-traumatic treatment methods and specialized oral hygiene practices.^43^ On the other hand, the thick biotype is characterized by greater resistance to physical trauma and gingival recession, resulting in better tissue management. This distinction underscores the importance of identifying gingival biotype to tailor treatment plans and achieve optimal clinical outcomes.^44^

 The results also revealed a significant correlation between GB and gender (*P* ≤ 0.05). Specifically, the thin GB was predominantly observed in women, while the thick biotype was more frequently found in men. Consistent with these findings, Bhat and Shetty^43^ and Anand et al.^44^ reported a significant relationship between GB and gender. Moreover, the study by Anand et al.^44^ reported that the thick GB was more prevalent in men, while the thin biotype was more common in women, further supporting the results of this study.

 Building on these findings, numerous studies have demonstrated that biological and physiological differences between genders play a pivotal role in determining GB.^45,46^ Specifically, hormones like estrogen in women can lead to thinner gingival tissue. In contrast, in men, higher connective tissue density and stronger blood flow typically result in a thicker biotype.^47,48^

 Additionally, the results showed that the mean age of patients with a thin biotype was significantly lower than that of patients with a thick biotype (*P* ≤ 0.05). In this context, Kolte et al.^49^ reported that in younger age groups, thinner gingival widths were more common, and women had thinner gingiva with narrower widths compared to men, consistent with the present study. Moreover, the most common reason for this difference is the higher density of collagen fibers and connective tissue in younger individuals. This greater collagen density provides more structural support, resulting in thicker gingiva. As age increases, collagen density decreases, leading to thinner gingival tissue.^50^

 In the present study, the KGW was significantly greater in the thick biotype compared to the thin biotype. Patients with a thin biotype had a narrower gingival width than those with a thick biotype. The study by Cook et al.^51^ found a positive correlation between GT and the width of keratinized tissue. They observed that thinner biotypes had a narrower zone of keratinized tissue, while thicker biotypes had a wider zone. These findings align with the present study, which also shows a significant relationship between GT and tissue width. In this regard, wider keratinized gingiva in thick biotypes offers greater resistance to mechanical forces and gingival recession. In contrast, thinner biotypes, due to less connective tissue support, have a narrower zone of keratinized gingiva. These structural differences in gingival tissue can explain the positive relationship between GT and keratinized tissue width.^13^

 In addition to soft tissue parameters, the influence of BBT must also be considered, as it plays a pivotal role in implant stability and esthetic outcomes. BBT has been widely recognized as a key determinant in implant planning and soft tissue stability.^52^ Aizcorbe-Vicente et al.^53^ stated that no minimum facial bone thickness was identified that would completely prevent peri-implant bone loss or ensure soft tissue stability; however, a thickness of approximately 2 mm has been associated with reduced vertical bone resorption and less mid-facial mucosal recession. When the buccal plate is thin, clinicians may need to consider guided bone regeneration^54^ and the use of narrower implants or adjustments in three-dimensional implant positioning to achieve long-term stability.^55‒57^

 Additionally, the suitability of flapless implant placement is strongly influenced by the quality and thickness of the buccal bone.^58^ Flapless surgery is more predictable when the buccal plate is intact and sufficiently thick, as the preservation of periosteal blood supply can minimize crestal remodeling.^59^ In contrast, sites with thin facial bone often require flap elevation and simultaneous bone grafting to prevent dehiscence, exposure of implant surfaces, and soft tissue complications.^60,61^ Furthermore, inadequate BBT has been consistently associated with a higher risk of mid-facial mucosal recession.^62^ Thin biotype patients with limited buccal bone support benefit from bone augmentation and, when indicated, soft tissue grafting to enhance peri-implant tissue volume and ensure more predictable esthetic outcomes.^61^

 Regarding PH, this study observed no statistically significant difference in mean PH between the thick and thin GBs (*P* > 0.05). Fischer et al.^63^ reported no statistically significant difference between PH and gingival biotype (thin and thick), whereas Malhotra et al.64 reported a significant difference. The findings of this section of the study align with Fischer et al.’s ^63^ study but do not correspond with those of Malhotra et al.^64^ The absence of a statistically significant difference in mean PH between thick and thin biotypes suggests that PH may be influenced more by other factors, such as underlying bone structure and interdental spacing, rather than directly correlating with GB thickness.^65^ This is especially relevant for patients with healthy periodontal features and without significant bone loss, where the stability and integrity of the soft tissue likely have a greater impact on the papilla height.^66^

 This study had several limitations that should be acknowledged. First, although gingival thickness measurements were performed using a standardized transgingival probing method, the possibility of observer bias cannot be eliminated. Second, CBCT images have inherent resolution limitations, particularly when assessing ultra-thin buccal bone plates, which may affect the precision of thickness measurements. Finally, the cross-sectional design of this study does not allow for longitudinal evaluation of how the gingival biotype may influence long-term clinical outcomes.

## Conclusion

 This study demonstrated that a thicker gingival biotype is associated with greater buccal bone thickness, as indicated by CBCT findings in the studied population. Differences in gingival biotype distribution were observed across age and gender groups. No statistically significant difference in papilla height was found between gingival biotypes. Future studies are warranted to further investigate biological and genetic factors influencing gingival biotype variability.

## Competing Interests

 The authors declare that they have no competing interests regarding authorship and/or publications of this paper.

## Data Availability

 The data from the reported study can be obtained upon request from the corresponding author.

## Ethical Approval

 This study was conducted in compliance with the ethical principles outlined in the Declaration of Helsinki. The protocol was reviewed and approved by the Ethics Committee of Ilam University of Medical Sciences (Ethics code: IR.MEDILAM.REC.1402.055). Written informed consent was obtained from all participants before their inclusion. Confidentiality and anonymity were maintained, and participants were informed of their right to withdraw at any time without consequences.
